# Identification of Caveolae-Associated Protein 4 Autoantibodies as a Biomarker of Immune-Mediated Rippling Muscle Disease in Adults

**DOI:** 10.1001/jamaneurol.2022.1357

**Published:** 2022-06-13

**Authors:** Divyanshu Dubey, Grayson Beecher, M. Bakri Hammami, Andrew M. Knight, Teerin Liewluck, James Triplett, Abhigyan Datta, Surendra Dasari, Youwen Zhang, Matthew M. Roforth, Calvin R. Jerde, Stephen J. Murphy, William J. Litchy, Anthony Amato, Vanda A. Lennon, Andrew McKeon, John R. Mills, Sean J. Pittock, Margherita Milone

**Affiliations:** 1 Department of Neurology, Mayo Clinic College of Medicine, Rochester, Minnesota; 2 Department of Laboratory Medicine and Pathology, Mayo Clinic College of Medicine, Rochester, Minnesota; 3 Department of Neurology, Brigham and Women’s Hospital, Boston, Massachusetts; 4 Department of Immunology, Mayo Clinic College of Medicine, Rochester, Minnesota

## Abstract

**Importance:**

Immune-mediated rippling muscle disease (iRMD) is a rare myopathy characterized by wavelike muscle contractions (rippling) and percussion- or stretch-induced muscle mounding. A serological biomarker of this disease is lacking.

**Objective:**

To describe a novel autoantibody biomarker of iRMD and report associated clinicopathological characteristics.

**Design, Setting, and Participants:**

This retrospective cohort study evaluated archived sera from 10 adult patients at tertiary care centers at the Mayo Clinic, Rochester, Minnesota, and Brigham & Women’s Hospital, Boston, Massachusetts, who were diagnosed with iRMD by neuromuscular specialists in 2000 and 2021, based on the presence of electrically silent percussion- or stretch-induced muscle rippling and percussion-induced rapid muscle contraction with or without muscle mounding and an autoimmune basis. Sera were evaluated for a common biomarker using phage immunoprecipitation sequencing. Myopathology consistent with iRMD was documented in most patients. The median (range) follow-up was 18 (1-30) months.

**Exposures:**

Diagnosis of iRMD.

**Main Outcomes and Measures:**

Detection of a common autoantibody in serum of patients sharing similar clinical and myopathological features.

**Results:**

Seven male individuals and 3 female individuals with iRMD were identified (median [range] age at onset, 60 [18-76] years). An IgG autoantibody specific for caveolae-associated protein 4 (cavin-4) was identified in serum of patients with iRMD using human proteome phage immunoprecipitation sequencing. Immunoassays using recombinant cavin-4 confirmed cavin-4 IgG seropositivity in 8 of 10 patients with iRMD. Results for healthy and disease-control individuals (n = 241, including myasthenia gravis and immune-mediated myopathies) were cavin-4 IgG seronegative. Six of the 8 individuals with cavin-4 IgG were male, and the median (range) age was 60 (18-76) years. Initial symptoms included rippling of lower limb muscles in 5 of 8 individuals or all limb muscles in 2 of 8 sparing bulbar muscles, fatigue in 9 of 10, mild proximal weakness in 3 of 8, and isolated myalgia in 1 of 8, followed by development of diffuse rippling. All patients had percussion-induced muscle rippling and half had percussion- or stretch-induced muscle mounding. Four of the 10 patients had proximal weakness. Plasma creatine kinase was elevated in all but 1 patient. Six of the 10 patients underwent malignancy screening; cancer was detected prospectively in only 1. Muscle biopsy was performed in 7 of the 8 patients with cavin-4 IgG; 6 of 6 specimens analyzed immunohistochemically revealed a mosaic pattern of sarcolemmal cavin-4 immunoreactivity. Three of 6 patients whose results were seropositive and who received immunotherapy had complete resolution of symptoms, 1 had mild improvement, and 2 had no change.

**Conclusions and Relevance:**

The findings indicate that cavin-4 IgG may be the first specific serological autoantibody biomarker identified in iRMD. Depletion of cavin-4 expression in muscle biopsies of patients with iRMD suggests the potential role of this autoantigen in disease pathogenesis.

## Key Points

**Question:**

Is there an autoantibody biomarker of immune-mediated rippling muscle disease (iRMD)?

**Findings:**

In this cohort study, autoantibodies to caveolae-associated protein 4 (cavin-4) were identified and orthogonally validated in 8 of 10 patients with iRMD; results for all healthy and disease-control individuals were seronegative. Immunohistochemical studies demonstrated depletion of cavin-4 expression in biopsied iRMD skeletal muscle.

**Meaning:**

The findings suggest that seropositivity for cavin-4 IgG, the first specific serological biomarker discovered for iRMD, may support an autoimmune pathogenesis for this clinical and immunohistopathologic entity.

## Teaser

This cohort study evaluates caveolae-associated protein 4 as a potential biomarker of immune-mediated rippling muscle disease.

## Introduction

Immune-mediated rippling muscle disease (iRMD) is a rare myopathy characterized by abnormal muscle excitability. It is usually electrically silent, exhibiting wavelike muscle contractions (rippling) and percussion- or stretch-induced muscle mounding.^1^ In contrast to hereditary rippling muscle disease (hRMD), associated to date with pathogenic variants in caveolin-3 (*CAV3*) or, less frequently, cavin-1 (*CAVIN1*) genes, patients with iRMD lack a defined genetic defect.^2,3^ Responsiveness to immunotherapy supports an autoimmune pathogenesis. Muscle acetylcholine receptor antibody positivity with or without clinical myasthenia gravis (MG) has been reported in association with iRMD, further favoring its potential immune-mediated etiology.^4,5,6^

Immunohistopathological analysis of biopsied muscle of patients with iRMD demonstrates a mosaic pattern of caveolin-3 deficiency,^4^ while loss of sarcolemmal caveolin-3 immunoreactivity is more diffuse in most hRMD cases. Some iRMD biopsies demonstrate endomysial lymphocytic inflammatory exudate further supporting an immune-mediated pathogenesis.^5^ Furthermore, a repeated muscle biopsy from 1 individual with iRMD after complete recovery of muscle symptoms showed restored sarcolemmal caveolin-3.^4^

In this study, we used phage immunoprecipitation sequencing, an unbiased proteomic technology, to identify a novel serological biomarker of iRMD, IgG specific for caveolae-associated protein 4 (cavin-4). We assessed the clinical specificity of this biomarker by testing sera from patients with autoimmune myopathies, autoimmune neuromuscular junction disorders, and from healthy control individuals. We also demonstrated patchy loss of the putative autoantigen in biopsied muscle of patients with iRMD.

## Methods

### Standard Protocol Approvals, Registrations, and Patient Selection

The Mayo Clinic Institutional Review Board approved human specimen acquisition and a waiver of consent for retrospective review of patient clinical data obtained for serologic test validation (IRB #16-009814, 18-010637). All patients at the Mayo Clinic, Rochester, Minnesota, whose medical records were reviewed provided written consent for medical research, and relevant reporting guidelines were followed.^7^ Clinical and outcome details were abstracted from medical records. Archival sera from 10 patients with clinical diagnoses of iRMD^1^ (9 identified by searching Mayo Clinic medical records from January 1, 2000, to August 2, 2021, and the Mayo Muscle Pathology Laboratory database) were retrieved in the Mayo Clinic Neuroimmunology Laboratory. All 10 patients were evaluated by a neuromuscular specialist as having symptoms and signs of rippling muscle disease, specifically percussion and/or stretch-induced rolling movements across a muscle or muscle group (rippling) and percussion-induced rapid muscle contraction with or without percussion-induced muscle mounding without associated spontaneous muscle activity and motor unit action potentials detected by electromyography (electrically silent).^8,9^ An immune-mediated etiology was supported by 1 or more of the following: mosaic pattern of sarcolemmal caveolin-3 immunoreactivity on muscle biopsy, immunotherapy responsiveness, and lack of mutations in the *CAV3* and *CAVIN1* genes. Nine patients had undergone diagnostic muscle biopsy at Mayo Clinic. Caveolin-3 immunohistochemical study was performed on 8 biopsies, 7 of which revealed a mosaic pattern of sarcolemmal caveolin-3 immunoreactivity typical of iRMD. The *CAV3* gene was sequenced in 9 cases, and the *CAVIN1* gene was also sequenced in 3 of these 9 cases. No potentially pathogenic variants were found in these genes. One patient (patient 10) who lacked the mosaic caveolin-3 pattern on muscle biopsy was included because of a lack of mutations in both *CAV3* and *CAVIN1*. One patient (patient 7) with rippling muscle disease, coexisting ocular MG, and anti–acetylcholine receptor seropositivity was evaluated at Brigham and Women’s Hospital, Boston, Massachusetts, by 2 of the authors (D.D. and A.A.); serum was sent to Mayo Clinic for additional investigation. This patient’s genotype (*CAV3* and *CAVIN1*) was unknown and muscle biopsy not performed; however, the associated seropositive MG at the onset of muscle rippling, commonly described in the iRMD literature, favored an immune-mediated etiology of the rippling.

### Phage Immunoprecipitation Sequencing

Sera of patients with iRMD and control individuals were incubated with 10^10^ plaque-forming units per milliliter of the whole–human proteome phage-display library, and antibody-bound phage particles were isolated by protein G immunoprecipitation (D.D. and A.K.). Patient IgG-bound phage particles were eluted, and next-generation sequencing libraries were prepared using the Illlumina TruSeq Nano DNA library preparation kit with associated indexes. Prepared libraries were sequenced on the Illumina NovaSeq platform using an SP flow cell (Illumina). Sequenced reads were processed using an in-house developed bioinformatics pipeline (S.D.) to identify the putative autoantigen (eMethods in the Supplement).

### Confirmation and Verification of Putative Autoantigen

A putative novel antigen was validated by testing patient sera using a protein expression vector-transfected COS7 cell-based assay (CBA), Western blotting, human muscle lysate immunoprecipitation, indirect immunofluorescence on cryosectioned rat skeletal muscle, or all of these methods (eMethods in the Supplement). Archived sera from 124 disease-control individuals (20 with dermatomyositis, 22 with immune-mediated necrotizing myopathy, 56 with MG without evidence or report of muscle rippling, 20 with aquaporin-4 positive neuromyelitis optica, 3 with peripheral nerve excitability, and 3 with sporadic late onset nemaline myopathy) and 123 healthy control individuals were tested for putative autoantigen by COS7 CBA.

### Muscle Biopsy Histochemical and Immunohistochemical Studies

Nine of the 10 patients had a muscle biopsy and conventional histochemical studies. Full methodological details are in the eMethods in the Supplement. Eight patients’ biopsies were immunostained for caveolin-3 for diagnostic purposes. Additional immunohistochemical studies were performed on residual biopsy tissue (IRB 18-010637) for patients 1 through 6 and 9 through 10. No residual tissue was available for patient 8. Immunohistochemical analysis was done in comparison with healthy control individual muscle biopsies and 2 patients with hRMD due to *CAV3* mutations (c.99C>G, p.Asn33Lys; c.169G>A, p.Val57Met). All muscle biopsy slides were analyzed independently by 3 authors (G.B., T.L., and M.M.).

## Results

### Autoantigen Identification

Seven male individuals and 3 female individuals with iRMD were identified (median [range] age at onset, 60 [18-76] years). Seeking a putative autoantigen for iRMD, we initially screened sera from 3 patients (patient 2, patient 6, and patient 8) by whole–human proteome phage immunoprecipitation sequencing and identified candidate antigens by using a bioinformatics pipeline to process individual phage immunoprecipitation sequencing enrichment data. IgG in all 3 cases bound to peptides corresponding to different regions of the cavin-4 protein (eFigure 1 in the Supplement), with 3 patients having common peptide hits (fragments 5, 9, and 10). We used sera from 2 patients with iRMD (patient 3 and patient 7) for subsequent protein G magnetic bead capture and mass spectrometry analysis of a human muscle lysate preparation (eMethods in the Supplement).

### Clinical Specificity of Cavin-4-IgG

**Figure 1.**
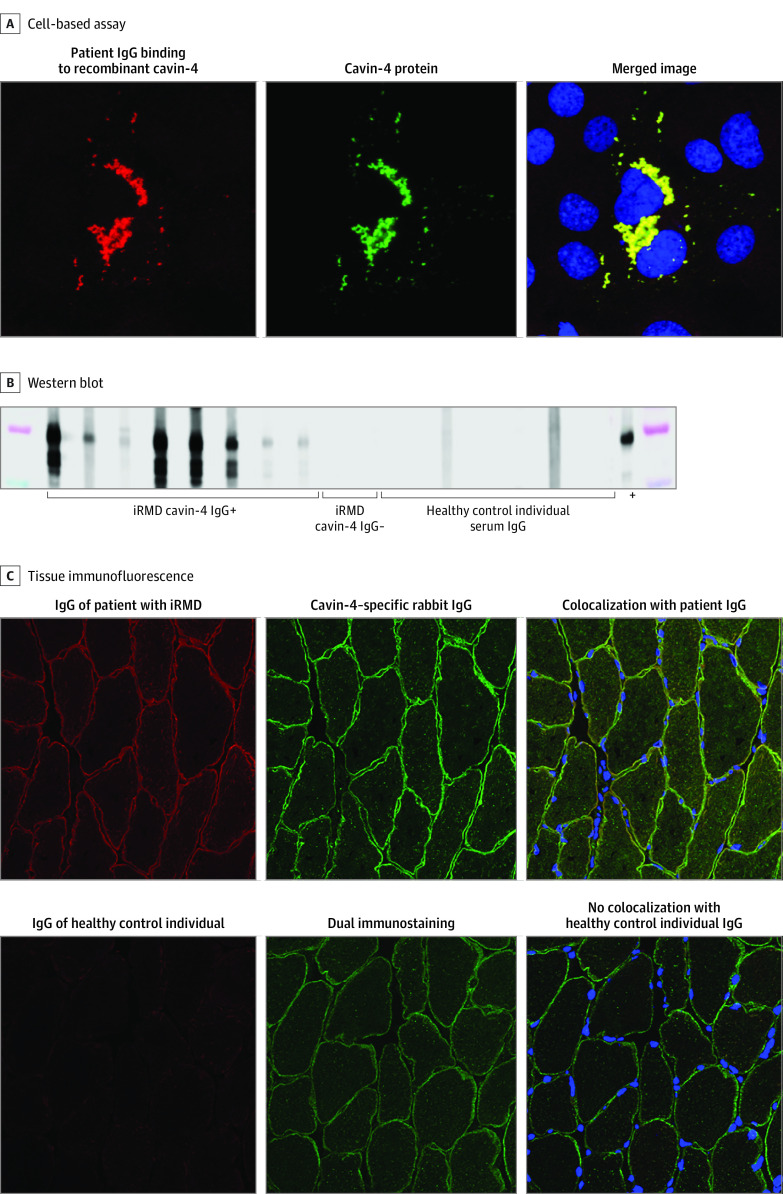
Validation of Caveolae-Associated Protein 4 (Cavin-4) IgG Specificity by Transfected Cell-Based Immunofluorescence, Western Blot, and Tissue-Based Immunofluorescence Assays A, Tetramethylrhodamine-conjugated anti–human IgG bound patient IgG demonstrated binding to recombinant cavin-4; green fluorescent protein–tagged cavin-4 protein expressed in the transiently transfected COS7 cells. B, Western blot of COS7 cell lysate containing recombinant cavin-4 protein demonstrated binding of IgG in the sera of 8 of the 10 patients with immune-mediated rippling muscle disease (iRMD) and a commercial cavin-4–specific rabbit IgG (designated +) to an approximately 70-kD protein; no healthy control individual serum IgG (N) bound. C, Dual immunostaining of cryosectioned rat skeletal muscle by a commercial cavin-4–specific rabbit IgG and by a patient IgG and healthy control individual IgG demonstrated colocalization with patient IgG (C3, merged image is yellow) but not with healthy control individual IgG (C6, merged image is green). Nuclei are stained blue by 4′,6-diamidino-2-phenylindole.

We next tested sera from all 10 patients with iRMD for cavin-4 IgG by immunofluorescent CBA, using cavin-4–transfected COS7 cells as substrate (eTable 2 in the Supplement). Results for 8 of the 10 individuals with iRMD (patients 1-8) were positive (Figure 1). IgG in 7 of the 8 with positive sera (patients 1-4 and 6-8) colocalized with a commercial cavin-4–specific IgG on rat skeletal muscle by immunostaining (Figure 1; eFigure 2 in the Supplement). Furthermore, IgG in all 8 patients’ sera yielded a positive band by Western blot on lysate containing recombinant cavin-4. IgG in 2 sera that were negative by CBA on transfected COS7 cell and Western blot on denatured cavin-4 protein (patients 9 and 10) were screened by whole–human proteome phage immunoprecipitation sequencing. IgG in 1 of those sera (patient 9) bound selectively to cavin-4 (with lower enrichment score than the initial 3 index sera) but the other serum (patient 10) was negative (eTable 1 in the Supplement).

The cavin-4–reactive IgG in all 8 positive sera was of IgG1 subclass. None of the sera from disease-control individuals (98 with immune-mediated myopathy or neuromuscular junction disorders and 20 with autoimmune CNS diseases) or 123 healthy control individuals contained cavin-4–reactive IgG. Furthermore, none of the 5 sera from patients with iRMD tested through phage immunoprecipitation sequencing contained IgG reactive with caveolin-3 or cavin-1, and none of the 10 sera from patients with iRMD tested by CBA (eMethods in the Supplement) were positive for caveolin-3 IgG.

### Clinical Characteristics of Patients With iRMD and Cavin-4 IgG 

Six of 8 patients with cavin-4 IgG were male. Initial symptoms included rippling of muscle in lower limbs in 5 of 8 patients or in all limbs, mild proximal weakness in 3 of 8 (Medical Research Council grade 4/5 in affected muscles), and isolated myalgia in 1 of 8. Diffuse rippling ensued. Fatigue was common (7 of 8 patients). Cardiac symptoms were absent. On examination, all patients had percussion-induced muscle rippling (Video), and half had percussion- or stretch-induced muscle mounding. Clinical comorbidities included type 2 diabetes in 1 patient, Hashimoto thyroiditis in 1, and pernicious anemia coexisting with lung biopsy-proven sarcoidosis in 1.

**Video.** Percussion-Induced Muscle Rippling in Patients With Caveolae-Associated Protein 4 (Cavin-4) IgG–Positive Immune-Mediated Rippling Muscle Disease (iRMD) Wavelike muscle rippling occurs perpendicular to the long axis of the quadriceps in response to percussion by the examiner’s hand in a patient with iRMD and cavin-4 IgG antibodies. Needle electromyography of the vastus lateralis (not shown) demonstrated electrical silence during the rippling, typical of this disorder.

Plasma creatine kinase was elevated in all but 1 patient (median [range], 512 [132-2625] U/L; normal range, 39-308 U/L in male individuals and 26-192 U/L in female individuals). Acetylcholine receptor–binding antibodies were detected in 4 of 8 individuals tested at diagnostic evaluation, and repetitive nerve stimulation on electrodiagnostic testing revealed a decrement of compound muscle action potential amplitude consistent with MG in 2 of those individuals; single-fiber EMG revealed markedly abnormal jitter in a third. Striational muscle autoantibodies were detected in 4 of 8 individuals (median [range] titer, 7680 [480-61 440]), concomitant with MG in patient 7 only. Cancer screening, performed in 6 of 8 individuals, included computed tomography (CT) of the chest, abdomen, and/or pelvis in 3 individuals, CT of the chest in 3 and ^18^F-fluorodeoxyglucose positron emission tomography and/or CT in 1. Breast carcinoma was detected in 1 patient (patient 6; previously described^6^) 6 months after iRMD symptom onset. Thymoma was not detected in any patient. Cardiac evaluation was performed in 3 patients: 12-lead electrocardiography results were normal in patient 1, demonstrated left bundle branch block in patient 6 with normal echocardiogram results, and demonstrated first-degree atrioventricular block in patient 9 with normal echocardiogram results.

### Muscle Histopathological and Immunohistochemical Features of Patients With iRMD and Cavin-4 IgG

**Figure 2.**
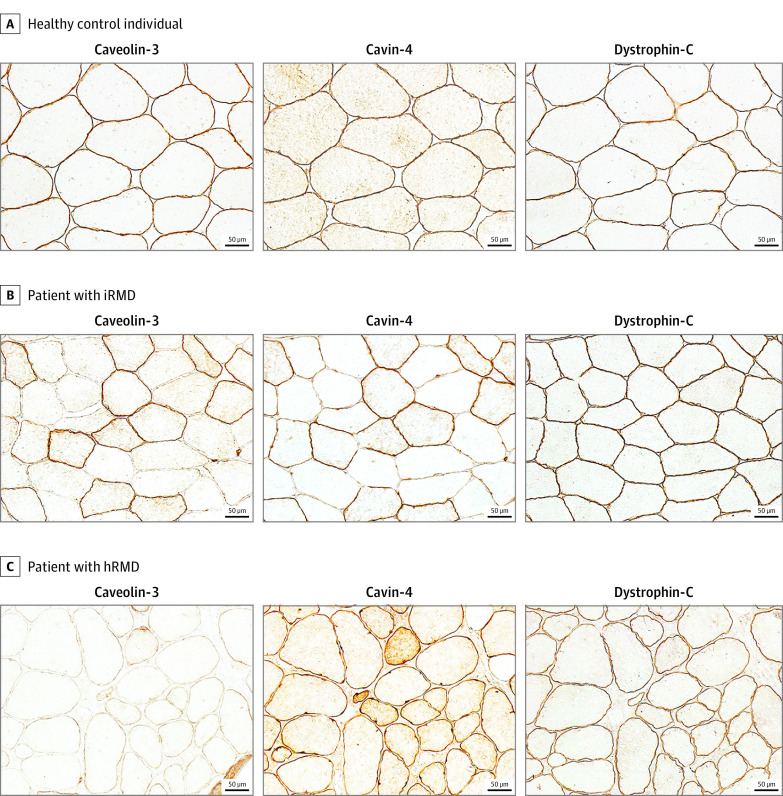
Caveolae-Associated Protein 4 (Cavin-4) and Caveolin-3 Muscle Immunohistochemistry Healthy control individual muscle sections demonstrate uniform sarcolemmal distribution of caveolin-3, cavin-4, and dystrophin immunoreactivities. Compared with healthy control individuals, muscle from a patient with immune-mediated rippling muscle disease (iRMD) displayed a mosaic pattern of sarcolemmal immunoreactivities for caveolin-3 and cavin-4 (a mixture of fibers with markedly attenuated or normal sarcolemmal immunoreactivity) but normal dystrophin immunoreactivity. Fibers with attenuated caveolin-3 and cavin-4 immunoreactivities were aligned on sequential sections. Muscle from a patient with hereditary rippling muscle disease (hRMD; *CAV3*, c.99C_>_G, p.Asn33Lys) demonstrated diffuse attenuation of sarcolemmal caveolin-3 immunoreactivity with preservation of cavin-4 and dystrophin.

Diagnostic muscle biopsy was performed in 7 of the 8 patients with cavin-4 IgG (patients 1-6 and 8). Immunohistochemical studies revealed in 6 of the patients tested (patients 1-6) a mosaic pattern of sarcolemmal caveolin-3 and cavin-4 immunoreactivity in contrast to the uniform sarcolemmal immunoreactivity for dystrophin-C-terminal peptide (Figure 2). In sequential sections, fibers with absent or attenuated caveolin-3 immunoreactivity matched those with attenuated cavin-4 immunoreactivity. The percentage of muscle fibers (per low power field) with deficient sarcolemmal caveolin-3 and cavin-4 immunoreactivity ranged from 55% to 91% (median 79%). A reduction in cavin-4 expression in muscle of patients whose results were seropositive was demonstrated additionally in muscle lysates by Western blot (eFigure 2 in the Supplement). Compared with iRMD muscle biopsies, hRMD muscle biopsies showed a diffuse attenuation (severe reduction or absence) of sarcolemmal caveolin-3 immunoreactivity but normal cavin-4 and dystrophin immunoreactivities (Figure 2). Immunohistochemical studies performed on healthy control individual muscle biopsies demonstrated normal sarcolemmal dystrophin, caveolin-3, and cavin-4 immunoreactivities (Figure 2).

**Figure 3.**
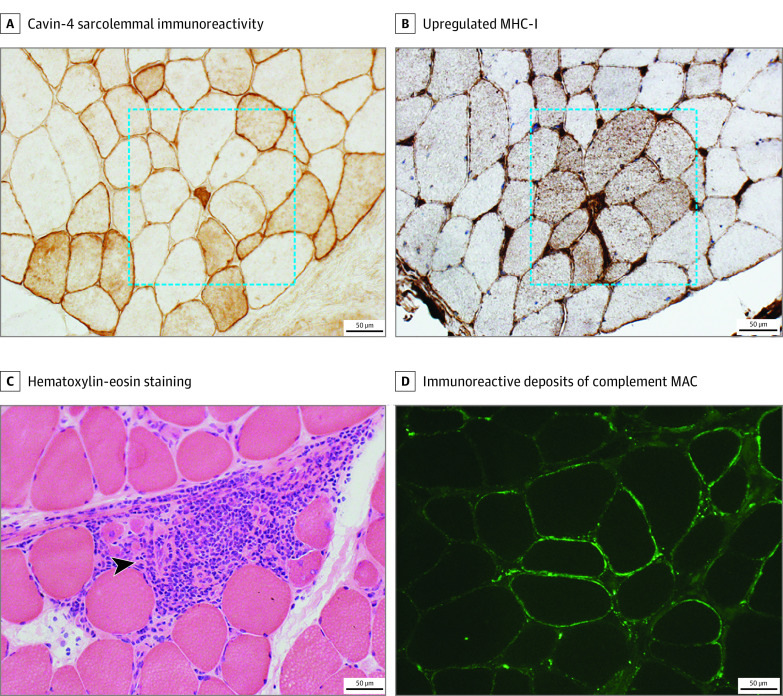
Inflammation, Major Histocompatibility Complex (MHC-I) Upregulation, and Complement Deposits Favor a Primary Immune-Mediated Pathogenesis for Immune-Mediated Rippling Muscle Disease Squares enclose fibers with marked attenuation of caveolae-associated protein 4 (cavin-4) sarcolemmal immunoreactivity and upregulated MHC-I. Hematoxylin-eosin–stained section demonstrates inflammatory cells (blue) in perimysium and endomysium and necrotic muscle fibers (arrowhead). Immunoreactive deposits of complement membrane attack complex (MAC) on the sarcolemma of scattered nonnecrotic fibers (area distant from the inflammatory reaction).

Of 7 individuals with iRMD, 2 had a muscle biopsy showing inflammation (Figure 3), which was described as minimal (minimal scattered inflammation) or mild (1 small collection per 5x-power field). Inflammation was localized to endomysial and perimysial perivascular regions in 1 individual or perimysial perivascular regions in 1. No autoaggressive inflammatory reaction was detected. Four biopsies had active myopathic features (muscle fiber necrosis and regeneration), which were minimal in 3 and moderate in 1.

A variable number of fibers in all iRMD muscle biopsies exhibited upregulation of sarcolemmal MHC-I and MAC deposition on nonnecrotic fibers (Table, Figure 3). Congo red stain did not detect amyloid deposition in any muscle biopsies, including those of the 2 patients with monoclonal gammopathy.

**Table.** Clinical, Laboratory, and Muscle Pathological Features of Patients With Immune-Mediated Rippling Muscle Disease

	P1	P2	P3	P4	P5	P6	P7	P8	P9	P10
Age at symptom onset, y	Mid-70s	Late teens	Mid-50s	Mid-40s	Mid-40s	Mid-70s	Mid-60s	Mid-40s	Early 50s	Early 60s
Presenting features^a^	Rippling quadriceps, myalgia, proximal weakness,^b^ fatigue	Diffuse rippling, percussion-induced muscle mounding, myalgia	Myalgia, fatigue	Rippling quadriceps, myalgia, fatigue	Diffuse rippling, myalgia, fatigue	Rippling quadriceps, myalgia, proximal weakness,^b^ fatigue	Rippling in lower limb muscles, fatigue	Rippling calves, proximal weakness,^b^ fatigue	Myalgia, fatigue	Myalgia, proximal weakness,^b^ fatigue
Follow-up, mo	16	24	6	2	19	22	1	29	30	6
Comorbidities at presentation	Hashimoto thyroiditis	None	Type 2 diabetes	None	None	Pulmonary sarcoidosis, pernicious anemia	None	None	None	Raynaud phenomenon
Concurrent MG	Yes	No	No	No	No	No	Yes	Yes	No	No
Cancer; screening performed	No; FDG-PET/CT body	No; none	No; CT chest, abdomen, pelvis	No; none	No; CT chest, abdomen, pelvis; testicular US; PSA	Breast carcinoma; CT chest abdomen, pelvis	No; CT chest	No; CT chest	No; CT chest, abdomen, pelvis	No; CT chest, FDG-PET/CT body
Thymoma	No	No	No	No	No	No	No	No	No	No
C3 sequencing	Normal	Normal	Normal	Normal	Normal	Normal	NP	Normal	Normal	Normal
C1 sequencing	NP	Normal	NP	NP	NP	NP	NP	NP	Normal	Normal
C4 IgG	Positive (≥1:6400)	Positive (≥1:6400)	Positive (1:6400)	Positive (1:1600)	Positive (≥1:6400)	Positive (1:3200)	Positive (1:3200)	Positive (≥1:6400)	Negative	Negative
C4 IgG1	Positive	Positive	Positive	Positive	Positive	Positive	Positive	Positive	Negative	Negative
CK, IU/L	885	2625	132	336	374	512	Unknown	534	566	1184
Muscle AChR IgG, nmol/L serum	5.94^c^	Negative	Negative	Negative	Negative	0.12	2.89^c^	6.31^c^	Negative	Negative
Other serum autoantibodies or IgG (value)	Titin, ANA, (titer 1280)	None	Titin, striational (titer, 7680)	Titin, striational (titer, 480)	IgG lambda MGUS (1.1 g/dL)	Titin, striational (titer, 61440)	Titin, striational (titer, 30720)	None	Titin, IgG kappa MGUS (0.7 g/dL)	P/Q-type VGCC (0.16 nmol/L)^d^
Muscle biopsied	Vastus lateralis	Vastus lateralis	Biceps brachii	Vastus lateralis	Vastus lateralis	Biceps brachii	NP	Vastus lateralis	Triceps	Supraspinatus
Inflammation, location	None	None	Rare^e^, perimysial perivascular	None	None	Mild^f^, endomysial & perimysial/ perivascular	NP	None	None	None
SC4 immunoreactivity pattern	Mosaic	Mosaic	Mosaic	Mosaic	Mosaic	Mosaic	NP	NP	Mosaic	Normal
Fibers lacking SC4 /10× LPF	63%	74%	84%	91%	89%	55%	NP	NP	18%	0%
Sarcolemmal C3 immunoreactivity pattern	Mosaic	Mosaic	Mosaic	Mosaic	Mosaic	Mosaic	NP	NP	Mosaic	Normal
Congruent C4 and C3 immunoreactivities	Yes	Yes	Yes	Yes	Yes	Yes	NP	NP	Yes	NA
MHC-I upregulation	+++^g^	+^h^	+^h^	++^i^	+^h^	+++^g^	NP	NP	+^h^	+^h^
Sarcolemmal MAC deposition	++^i^	++^i^	++^i^	+^h^	+^h^	+++^g^	NP	NP	++^i^	++^i^

Abbreviations: AChR, acetylcholine receptor; ANA, antinuclear antibody; C1, cavin-1; C3, caveolin-3; C4, cavin-4; CK, creatine kinase; CT, computerized tomography; FDG-PET, fluorodeoxyglucose-positron emission tomography; LPF, low-power field; MAC, membrane attack complex; MHC-I, major histocompatibility complex I; MG, myasthenia gravis; MGUS, monoclonal gammopathy of uncertain significance; NA, not applicable; NP, not performed; P, patient; PSA, prostate-specific antigen; SC4, sarcolemmal cavin-4; US, ultrasonography; VGCC, voltage-gated calcium channel.

^a^ All patients developed muscle rippling.

^b^ Medical Research Council grade 4/5 in weak muscles.

^c^ Disorder of neuromuscular transmission confirmed by electrodiagnostic testing (>10% decrement on repetitive nerve stimulation and/or abnormal single-fiber electromyography).

^d^ No evidence of a presynaptic or postsynaptic defect of neuromuscular transmission by repetitive nerve stimulation and single-fiber electromyography.

^e^ Minimal scattered inflammation.

^f^ One small collection per 5×-power field.

^g^ ≥2 Positive fibers per 10×-power field.

^h^ >3 Positive fibers per biopsy, <1 positive fiber per 10×-power field.

^i^ ≥1 Positive fibers per 10×-power field, <2 positive fibers per 10×-power field.

### Treatment and Outcomes of Patients With iRMD and Cavin-4 IgG

Immunotherapy with 1 or more agents was used in the illness course in 6 of 8 individuals, the regimens being individualized by clinician preference: intravenous immune globulin (IVIG) in 4, oral prednisone in 4, intravenous methylprednisolone in 1, plasmapheresis in 1, and azathioprine in 2, both with MG. The previously reported patient with breast cancer (patient 6) was treated with lumpectomy, regional radiation, and tamoxifen in addition to IVIG, 2g/kg monthly, for 2 cycles and prednisone, 50 mg tapering to 5 mg over 6 months, with complete remission of muscle rippling and weakness.^6^ At last follow-up, 2 additional patients had achieved complete remission (patient 1 within 4 months of treatment with prednisone, 20 mg daily; IVIG, 2g/kg monthly; and azathioprine, 2.5 mg/kg daily and patient 8 within 6 months of treatment with prednisone, 20 mg daily, and azathioprine, 2.5 mg/kg daily). Rippling was mildly improved in patient 3 (within 6 months of 1 course of plasmapheresis followed by 2 months of IVIG, 0.4g/kg weekly, then 3 months of intravenous methylprednisolone, 500 mg weekly). Two patients were immunotherapy refractory but clinically stable (patient 7 had coexisting MG, which improved considerably with prednisone, up to 50 mg daily, followed by gradual taper while rippling stabilized; patient 2 received only IVIG, 0.4 g/kg, biweekly for 2 years without marked improvement, declining other immunotherapies thereafter).

### Patients With iRMD and Without Cavin-4 IgG

Neither of the 2 patients without cavin-4 IgG (patients 9 and 10) had a variant in the *CAV3* or *CAVIN1* genes. They had both presented with myalgia and fatigue, and patient 10 had proximal weakness. Diffuse muscle rippling (sparing bulbar muscles) developed in both patients, and both had percussion-induced muscle rippling and muscle mounding. Neither had electrophysiological evidence of a neuromuscular transmission defect or radiological evidence of cancer.

Only patient 9 had a mosaic pattern of cavin-4 and caveolin-3 immunostaining with a low percentage (18%) of muscle fibers showing attenuation of cavin-4 sarcolemmal immunoreactivity, and cavin-4-IgG was detected in this patient by retrospective phage immunoprecipitation sequencing but not by other validation studies. Neither muscle biopsy showed an inflammatory exudate, but 1 had rare necrotic and regenerating muscle fibers, and both had patchy upregulation of sarcolemmal MHC-1 and MAC deposition. In support of an immune-mediated basis, patient 9 experienced complete symptom remission within 1 month after intravenous methylprednisolone, 500 mg weekly for 4 weeks, with maintenance of IVIG, 0.4 g/kg, and azathioprine, 2 mg/kg, daily thereafter. In the year prior to evaluation at our institution, patient 10 received IVIG, 2g/kg over 5 days, then monthly, 1g/kg, for 2 courses without improvement; the patient has remained clinically stable taking monthly IVIG, 2g/kg, over short-term follow-up.

## Discussion

Using phage immunoprecipitation sequencing, we identified cavin-4 IgG as a specific serological biomarker of iRMD in patients who were characterized clinically and, in most cases, had histopathological findings typical of iRMD. Results for control patients with MG, immune-mediated myopathies, or other autoimmune diseases and healthy control individuals were uniformly seronegative. One of the patients with cavin-4 IgG-had breast adenocarcinoma in a time course consistent with the iRMD having a paraneoplastic etiology. Malignancy screening revealed no tumor in the remaining patients screened. However, owing to limited duration of follow-up, we could not entirely exclude a paraneoplastic etiology. Three patients had a concurrent diagnosis of MG, and a fourth had seropositive results for acetylcholine receptor autoantibodies but lacked clinical and electrodiagnostic evidence of a neuromuscular transmission defect. Patchy sarcolemmal deficiency of cavin-4 immunoreactivity in more than 50% of muscle fibers was documented in all patients with cavin-4 IgG with available muscle histopathology. Reduction in muscle cavin-4 expression was previously demonstrated in a single patient with mosaic caveolin-3 expression,^10^ but it was not established whether that patient had an underlying *CAV3* mutation or met diagnostic criteria for iRMD. Cavin-4 is abundant in cardiac muscle as well as skeletal muscle.^11^ Even though none of the patients in our study reported cardiac symptoms, subclinical cardiac involvement cannot be entirely excluded as cardiac evaluation was limited.

iRMD specific autoantibodies have long been sought.^12,13,14^ Walker et al^12^ reported detection of autoantibodies directed against muscle proteins of high molecular weight (approximately 400 kD) and moderate molecular weight (approximately 97 kD) in sera of 3 patients with iRMD who had coexisting MG, but a specific autoantigen was not identified. In 2006, those investigators used a lambda phage human skeletal muscle cDNA library (Stratagene) to screen sera from individuals with iRMD and detected a striational antigen, titin isoform N2A, in 5 of 11, ATP synthase 6 in 1 of 11, and protein phosphatase 1 regulatory subunit 3 in 1 of 11.^13^ Although previously described in patients with iRMD,^15^ striational autoantibodies are not restricted to iRMD^16^ and, accordingly, in the report by Walker et al,^12^ IgG reactive with titin isoform N2A was also detected among MG control patients.^13,14^ An IgG reactive by Western blot with a protein approximately 43 kD in denatured control skeletal muscle was reported by Lo et al^4^ in sera from 2 patients with iRMD. Actin and rapsyn were excluded as the 43kD antigen, but a specific autoantigen was not identified.

Immunotherapy received prior to serum sampling is a plausible explanation for the cavin-4 IgG seronegativity in patients with iRMD, both of whom lacked mutations in *CAV3* and *CAVIN1*. For patient 9, the subsequent resolution of symptoms, the muscle biopsy’s mosaic pattern of caveolin-3 loss, and the detection of cavin-4–binding IgG in the serum by phage immunoprecipitation sequencing (but not by other methods) strongly supported an autoimmune pathogenesis. Follow-up information is limited for patient 10. The age at onset would favor an acquired etiology, but the patient’s response to immunotherapy was equivocal and the muscle biopsy lacked the mosaic pattern of caveolin-3 loss characteristic of iRMD. An underlying defect in a gene not yet known to cause muscle rippling cannot be excluded. It remains possible that an autoantibody different from cavin-4 IgG may be associated with iRMD in these cases.

Cavin-4 has been demonstrated in zebrafish to play a role in structural and functional maturation of T-tubules.^17^ In the absence of cavin-4, T-tubules fail to lose caveolar components and remodel, leading to fragmentation of the T-tubules and functional defects in calcium ion release. This in turn may induce abnormal muscle excitability, especially to stretch or pressure. Therefore, anti–cavin-4 antibody–mediated disruption could affect the stability of the T-tubule membrane and the excitation-contraction coupling system.^18^ As cavin-4 is expressed in the T-tubule membrane, one can speculate that the binding of patient IgG to the protein in vivo may cause antibody or complement mediated protein depletion. Despite the depletion of caveolin-3 immunoreactivity in muscle biopsies of all patients with cavin-4 IgG, whole–proteome phage immunoprecipitation sequencing or caveolin-3 CBA did not reveal IgG specific for caveolin-3. Loss of caveolin-3 from the T-tubules of the muscle of patients with iRMD is presumably secondary to its interaction with cavin-4,^19^ as it has been demonstrated in autoimmune neuromyelitis optica to account for loss of the excitatory amino acid transporter-2 from astrocytes secondary to the noncovalent interaction of aquaporin-4 with excitatory amino acid transporter-2.^20^ The mechanisms leading to abnormal muscle excitability remain to be elucidated. Clustering of caveolin-3 in areas crucial for propagation of the contractile impulse suggests its loss could alter muscle fiber mechanofunction by impeding action potential propagation through a defective T-tubule system.^21^

It remains to be determined whether in-vivo binding of cavin-4–specific IgG to myofibers accelerates the degradation of cavin-4 or activates the cytolytic complement cascade. The finding that IgG1 subclass predominated among cavin-4–reactive IgGs in all patients with iRMD whose results were seropositive suggests that complement may be an important effector of iRMD pathogenicity, as is known for MG and neuromyelitis optica.^22,23^ Although the finding of terminal complement components deposited on nonnecrotic muscle fibers supports a role for IgG-initiated complement activation in iRMD, MAC deposition is not specific and occurs also in muscular dystrophies.^24,25^ The finding of lymphocytic infiltration in a few muscle biopsies may be indicative of an autoantigen-specific T cell–mediated immune response contributing to cavin-4 depletion early in the disease course.^26^ Focused investigation of the immunopathogenesis of iRMD in terms of cavin-4 protein will require additional studies, such as in-vitro studies using live myotubes and patient IgG, patient peripheral blood T cell responses to cavin-4 peptides, and animal models of cavin-4 autoimmunity.

### Limitations

This study has limitations, including its retrospective design for data collection. In some cases, serum was collected after initiation of immunotherapy. In addition, 1 patient (patient 7) with cavin-4 IgG did not undergo genetic testing to search for *CAV3* or *CAVIN1* pathogenic variants.

## Conclusion

To our knowledge, cavin-4 IgG is the first specific serological autoantibody biomarker identified in iRMD. Phage display was used to identify this novel autoantibody. Depletion of cavin-4 expression in the muscle biopsies of patients with iRMD suggests the potential role of this autoantigen in disease pathogenesis.

## Notes

Supplement. eMethods eFigure 1. Discovery of cavin-4 autoantibodies in immune mediated rippling muscle disease patient sera by phage immunoprecipitation sequencing eFigure 2. Reduced cavin-4 expression in seropositive patient muscle biopsies eTable 1. Summary of methods by which autoantibodies to cavin-4 were identified in each patient eTable 2. Immune mediated rippling muscle disease and disease controls tested on cavin-4 CBA
