# Supplementary material for: Identification of Caveolae-Associated Protein 4 Autoantibodies as a Biomarker of Immune-Mediated Rippling Muscle Disease in Adults
Source: JAMA Neurol. 2022 Jun 13;79(8):808–16. doi: 10.1001/jamaneurol.2022.1357 (PMC9361081; doi:10.1001/jamaneurol.2022.1357)
Supplement: Supplement. — eMethods eFigure 1. Discovery of cavin-4 autoantibodies in immune mediated rippling muscle disease patient sera by phage immunoprecipitation sequencing eFigure 2. Reduced cavin-4 expression in seropositive patient muscle biopsies eTable 1. Summary of methods by which autoantibodies to cavin-4 were identified in each patient eTable 2. Immune mediated rippling muscle disease and disease controls tested on cavin-4 CBA [file jamaneurol-e221357-s001.pdf]

Supplemental Online Content

Dubey D, Beecher G, Hammami MB, et al. Identification of caveolae-associated protein 4 autoantibodies as a biomarker of immune-mediated rippling muscle disease in adults. *JAMA Neurol*. Published online June 13, 2022. doi:10.1001/jamaneurol.2022.1357

eMethods

eFigure 1. Discovery of cavin-4 autoantibodies in immune mediated rippling muscle disease patient sera

eFigure 2. Reduced cavin-4 expression in seropositive patient muscle biopsies

eTable 1. Summary of methods by which autoantibodies to cavin-4 were identified in each patient

eTable 2. Immune mediated rippling muscle disease and disease controls tested on cavin-4 CBA

This supplemental material has been provided by the authors to give readers additional information about their work.

© 2022 Dubey D et al. *JAMA Neurology.*

eMethods

Phage immunoprecipitation sequencing bioinformatics

The bioinformatics method started by quality assessing the raw read data of each sample using FASTQC software.[1] Reads were then mapped against a custom-built reference containing the 150bp-length oligo sequences, each representing a peptide, using bwa-mem software configured with default parameters.[2] An in-house developed program written in AWK programming language processed the aligned data and enumerated the total number of reads that perfectly mapped (i.e. full length match with no sequence modifications) to each peptide. Sample-wise peptides and their respective read counts were loaded into R programming environment (version 4.0.1) to generate enrichment scores. This process started by generating the mean read count observed for each peptide in control samples. A peptide enrichment score was computed for each (sample, peptide) pair by taking the ratio of counts observed in that pair to the mean count observed in control samples. A protein level enrichment score was computed by summing the enrichment scores observed for all of its peptide hits across all patient samples. Proteins were then ranked by the descending order of their enrichment scores and top 20 proteins were considered for further evaluation.

Cavin-4 cell-based assay (CBA)

Autoantigen specificity was confirmed by indirect immunofluorescence on COS7 cells transfected with green fluorescent protein (GFP)-tagged human with cavin-4-expressing plasmid. Cells were fixed (4% paraformaldehyde in PBS, 15 minutes), permeabilized (0.5% Triton X-100 in PBS, 2 minutes), and blocked for 1 hour with normal goat serum

© 2022 Dubey D et al. *JAMA Neurology.*

(10% in PBS). After PBS wash, cells were incubated for 40 minutes with patient serum (1:200 dilution), or rabbit cavin-4 specific IgG (1:200). After PBS wash and incubation with secondary antibodies (1:200 tetramethylrhodamine [TRITC]-conjugated goat antirabbit IgG and goat antihuman IgG; SouthernBiotech, Birmingham, AL). Coverslips were mounted using ProLong Gold antifade medium (containing 4,6-diamidino-2-phenylindole [DAPI]; Molecular Probes, Thermo Fisher Scientific). All assays were scored by at least two independent reviewers (DD, MBH). Confocal images were captured using a microscope (×20 or ×40 water immersion lens, LSM710; Carl Zeiss, Oberkochen, Germany). Patients seropositive for cavin-4 IgG were also tested for cavin-4 IgG subclasses using subclass-specific secondary antibodies (mouse anti-human IgG1-4 Fc-specific FITC-conjugated [Southern Biotech]).

Cavin-4 immunofluorescence assay (IFA)

Patient serum and commercial rabbit antibodies were tested on a cryosectioned composite of adult rat skeletal muscle sections. Sections were fixed using 4% paraformaldehyde for 1 minute, then permeabilized with 3-([3-cholamidopropyl] dimethylammonio)-1-propanesulfonate, 0.5%, in PBS for 1 minute, and then blocked for 1 hour with normal goat serum (10% in PBS). After PBS rinse, patient specimen (diluted 1:240) combined with commercial cavin-4 IgG (1:100, Invitrogen) was applied. After 40 minutes, and PBS wash, a human-specific secondary antibody conjugated with FITC (Scimedx Corporation, Denville, NJ), combined with a rabbit-specific secondary antibody conjugated with TRITC (1:200, Southern Biotech) was applied. Cover slips were mounted using ProLong Gold antifade medium (containing DAPI; Molecular

© 2022 Dubey D et al. *JAMA Neurology.*

Probes, Thermo Fisher Scientific). Confocal images were captured using a microscope (×20 or ×40 water immersion lens, LSM710; Carl Zeiss).

Western Blot

Fourty hours following transfection with cavin-4-GFP plasmid, cells were washed once in cold 1X PBS and one mL of cold RIPA (50 mM TrisHCL pH 7.5, 150mM NaCL, 1.0% Triton-X, 0.1% SDS, Roche protease inhibitor tablet) was added. Cells were scrapped off the dish, lysates were homogenized and allowed to lyse in RIPA for an additional 30 minutes at 4 degrees with gentle agitation. Lysates was used for Western blotting (separated in 10% polyacrylamide; transblotted to nitrocellulose membranes), IgG probes were from healthy controls (1:200), candidate patients (1:200), or commercial cavin-4 specific IgG (1:1000).

Caveolin-3 cell-based assay

COS7 cells transfected with green fluorescent protein (GFP)-tagged human with caveolin-3-expressing plasmid were fixed (4% paraformaldehyde, 15 minutes), permeabilized (0.5% Triton X-100, 2 minutes), and blocked for 1 hour with normal goat serum (10% in PBS). After PBS wash, cells were incubated for 40 minutes with patient serum (1:200 dilution), or rabbit caveolin-3 specific IgG (1:200). After PBS wash and incubation with secondary antibodies (1:200 tetramethylrhodamine [TRITC]-conjugated goat antirabbit IgG and goat antihuman IgG; SouthernBiotech, Birmingham, AL). Coverslips were mounted using ProLong Gold antifade medium (containing 4,6-diamidino-2-phenylindole [DAPI]; Molecular Probes, Thermo Fisher Scientific). Assay were scored by at least two independent reviewers (DD, AD).

© 2022 Dubey D et al. *JAMA Neurology.*

Titin immunoblots

EUROLINE PNS 12 Ag (Euroimmun) was utilized to evaluate for titin seropositivity. According to the manufacturer's instructions, samples were considered positive when presenting intensity was equal or above 15. All iRMD patients with available sera (all patients, except P8) were tested for the titin IgG (positive results reported in Table 1). We additionally tested 18 cavin-IgG negative and striational antibody myasthenia gravis patients, 16 of whom were positive for titin IgG.

Human muscle lysate immunoprecipitation sequencing

For immunoprecipitation, 1ul of patient's IgG and control normal sera IgG was exposed to 1 ml of human muscle lysate preparation for 1 hour then separately complexed to 10 ul protein G magnetic beads (Dynabeads; Invitrogen, Carlsbad, CA; Thermo Fisher Scientific, Waltham, MA) for 30 minutes (DD, MBH, AK). After washing, beads were boiled for 10 minutes in 2 × sample buffer. Eluted sample was electrophoresed using a 5 to 15% polyacrylamide gel, and proteins were located by silver staining (Bio-Rad Laboratories, Hercules, CA) and by Western blot. The immunoreactive bands specific to patient IgG immunoprecipitation was excised from the stained gel, reduced, alkylated with iodoacetamide, and digested with trypsin. Peptides in the digested sample were analyzed using nano high-pressure liquid chromatography electrospray tandem mass spectrometry (Mayo Clinic Medical Genome Facility, Proteomics Core).

Muscle biopsy histochemical and immunohistochemical studies

Conventional histochemical studies were performed on fresh frozen muscle biopsy 10-µm thick sections and stained for hematoxylin-eosin, modified Gomori trichrome, NADH

© 2022 Dubey D et al. *JAMA Neurology.*

dehydrogenase, succinate dehydrogenase, cytochrome c oxidase, acid phosphatase, adenosine triphosphatase (ATPase; at pH 4.3, 4.6, and 9.4), toluidine blue ATPase, phosphorylase, periodic acid–Schiff, oil red O, non-specific esterase, and Congo red. For immunohistochemical studies, Muscle sections were fixed in -20°C acetone for 10 minutes. Non-specific binding was blocked by pre-incubation with 2% PBS-BSA containing 10% donkey (for cavin-4 immunostained sections) or goat serum (for caveolin-3 and dystrophin immunostained sections). For immunohistochemical studies, 10-micron thick consecutive frozen sections were reacted with monoclonal IgGs specific for caveolin-3 (610421, BD Transduction Laboratories, San Diego, CA), cavin-4 (HPA020973, Sigma-Aldrich, St. Louis, MO), dystrophin C-terminal (control protein, NCL-DYS2, Novocastra, Leica Biosystems, Buffalo Grove, IL), major histocompatibility complex I (MHC-I; M073601-2, Agilent Technologies, Santa Clara, CA) and membrane attack complex (MAC [mouse anti-human C5b-9]; M077701-5, Agilent Technologies, Santa Clara, CA).[9] Cavin-4 , caveolin-3 , and dystrophin C-terminal proteins were localized with the immunoperoxidase method, using a biotinylated secondary antibody and the ABC kit (PK-6100; Vector Laboratories, Burlingame, CA) and diaminobenzidine (DAB) working solution (K3468; Dako,Carpinterin, CA) . Muscle section incubation with primary antibodies was performed overnight at 4°C, and subsequently with biotinylated secondary antibodies for 45 minutes at room temperature. For MHC-I immunohistochemical studies, sections underwent protein blocking (RE7150-K; NovoLink Polymer Detection System, Novocastra, Leica Biosystems, Buffalo Grove, IL) for 5 minutes followed by PBS rinse for 5 minutes twice and subsequent incubation with the primary MHC-I antibody for 60 minutes. Then followed the suggested procedure of NovoLink Polymer Detection System to finalize immunostaining.

© 2022 Dubey D et al. *JAMA Neurology.*

All muscle biopsies were assessed the following parameters: presence, size, and distribution of inflammatory exudate; pattern of caveolin-3 and cavin-4 sarcolemmal immunoreactivity (normal, mosaic, or absent), percentage of fibers lacking caveolin-3 and cavin-4 sarcolemmal immunoreactivity per low-power field (LPF, 10x magnification, averaged across four randomly selected LPFs), caveolin-3 sarcolemmal immunoreactivity pattern matching cavin-4 immunoreactivity. Number of fibers per LPF displaying sarcolemmal MHC-I immunoreactivity and number of non-necrotic fibers displaying sarcolemmal MAC immunoreactivity.

Patient muscle western blot

Frozen patient muscle tissue biopsy was washed once in cold 1X PBS and 400ul of cold RIPA (50 mM TrisHCL pH 7.5, 150mM NaCL, 1.0% Triton-X, 0.1% SDS, Roche protease inhibitor tablet) was added. The now thawed tissue was homogenized periodically over 30 minutes while maintained on ice. Protein concentrations were obtained using BCA protein assay (Pierce, Thermo Scientific). For each muscle sample, 2 lanes, each containing 10 µg of the protein in SDS PAGE sample buffer with B mercaptoethanol were run on a 4 -15% SDS-PAGE gel, followed by transfer of the protein to nitrocellulose paper. Each muscle sample was then subjected to rabbit polyclonal anti-MURC/cavin-4 antibody (HPA020973, Sigma-Aldrich) at a concentration of 1:1000 on one lane and rabbit polyclonal antibody to GAPDH at a concentration of 1:2500 on the other lane, followed by goat HRP-conjugated anti-rabbit secondary antibodies (1:2000).

© 2022 Dubey D et al. *JAMA Neurology.*

eFigure 1. Discovery of cavin-4 autoantibodies in immune mediated rippling muscle disease patient sera

Immunoprecipitation of human proteome phage display library (10^{10} plaque-forming units [PFU] per milliliter) by IgG in sera from iRMD patients (IP1-5) and healthy controls (IP6, IP7). Cavin-4 protein was bound by IgG in four of the five patients' sera (IP1-4, IP4 was negative when tested by validation methodology, therefore considered seronegative) but not by one patient's serum (IP5, patient P10) nor by healthy control sera (A). Note common enrichment of cavin-4 oligopeptide fragments 5, 9 and 10 by IgG in iRMD patients' sera (B). Peptide sequence of fragments 5, 9 and 10 are bold and underlined (C).

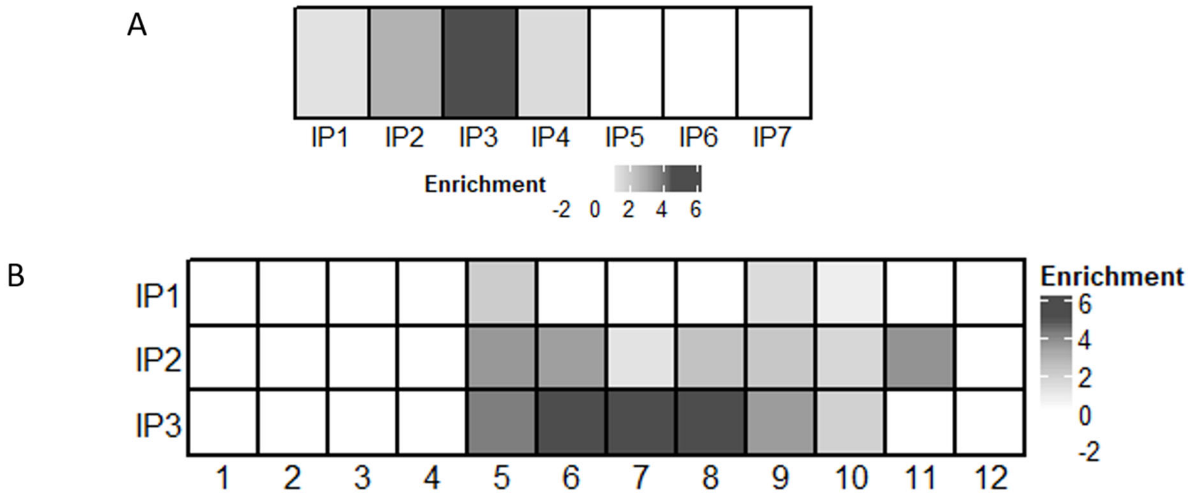

C

1 mehngsasna dkihqnrlss vtededqdaa ltivtvldkv asivdsvqas qkrieerhre
61 menaiksvqi dllklsqshs ntghiinklf ektrkvsahi kdvkarvekq qihvkkvevk
121 qeeimkknkf rvvifqekfr cpt**slsvvkd rnltenqeed dddifdppvd lssdeeyyve**
181 **esrsarlrks gke**hidnikk afskenmqkt rqnldkkvnr irtrivtper rerlrqsger
241 lrqsgerlrq sgerfkksis naapskeafk mrslrkgkdr tvaegee**car emgvdiiars**
301 **eslgpisely sdelsepehe aarpvypphe greiptp**epl kvtfksqvkv eddesllldl
361 khss

© 2022 Dubey D et al. *JAMA Neurology.*

eFigure 2. Reduced cavin-4 expression in seropositive patient muscle biopsies

Western blot demonstrating reduced cavin-4 protein concentration in the muscle biopsies of cavin-4 IgG seropositive patients (P1-6; Lane 1-6), compared to cavin-4 seronegative IRMD cases (Lane 7-8). All patients except for patient 6 (dashed box) had relatively similar GAPDH concentrations supporting equitable muscle protein loading.

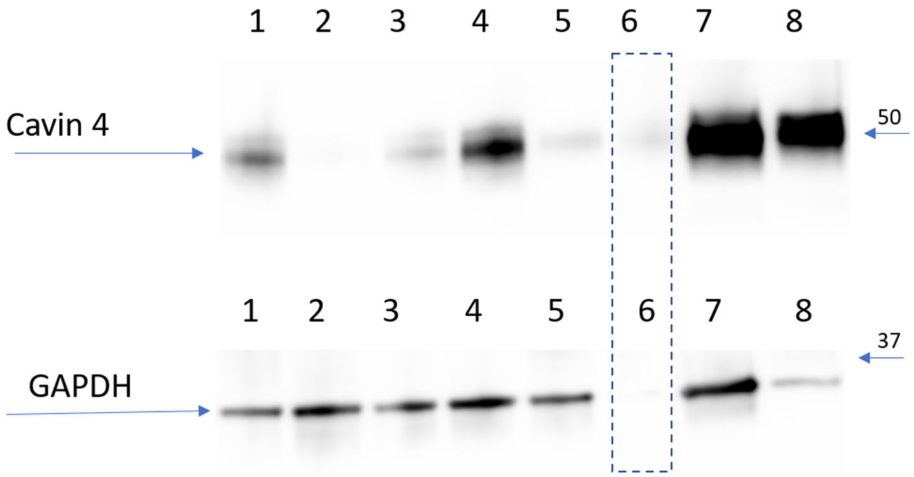

1	P1	Cavin-4 IgG positive iRMD
2	P2	Cavin-4 IgG positive iRMD
3	P3	Cavin-4 IgG positive iRMD
4	P4	Cavin-4 IgG positive iRMD
5	P5	Cavin-4 IgG positive iRMD
6	P6	Cavin-4 IgG positive iRMD
7	P9	Cavin-4 IgG negative iRMD
8	P10	Cavin-4 IgG negative iRMD

© 2022 Dubey D et al. *JAMA Neurology.*

eTable 1. Summary of methods by which autoantibodies to cavin-4 were identified in each patient

Patient	Patient Phage Display	Cell-Based Overexpression Assay	Overexpression Cell Lysate Western blot	Rodent Skeletal muscle Immunohistochemistry
1	Not tested	Positive	Positive	Positive
2	Positive	Positive	Positive	Positive
3	Not tested	Positive	Positive	Positive
4	Not tested	Positive	Positive	Positive
5	Not tested	Positive	Positive	Negative
6	Positive	Positive	Positive	Positive
7	Not tested	Positive	Positive	Positive
8	Positive	Positive	Positive	Positive
9	Positive	Negative	Negative	Negative
10	Negative	Negative	Negative	Negative

© 2022 Dubey D et al. *JAMA Neurology.*

eTable 2. Immune mediated rippling muscle disease and disease controls tested on cavin-4 CBA

Samples tested	Cavin-4 CBA positive/total
Immune mediated rippling muscle disease	8/10 (serum)
Dermatomyositis	0/20 (serum)
Immune-mediated necrotizing myopathy	0/22 (serum)
MG without evidence/report of muscle rippling	0/56 (serum)
Aquaporin-4 positive NMO	0/20 (serum)
Cases with peripheral nerve hyperexcitability (2 with Morvan's syndrome)	0/3 (serum)
SLONM	0/3 (serum)
Normal controls	0/123 (serum)

Key: MG, myasthenia gravis; NMO, neuromyelitis optica; SLONM, Sporadic late onset nemaline myopathy.

© 2022 Dubey D et al. *JAMA Neurology.*

References

1. Wingett SW, Andrews S. FastQ Screen: A tool for multi-genome mapping and quality control. *F1000Res.* 2018;7:1338.
2. Li H, Durbin R. Fast and accurate short read alignment with Burrows-Wheeler transform. *Bioinformatics.* 2009;25(14):1754-1760.

© 2022 Dubey D et al. *JAMA Neurology.*
